# Make the Invisible Visible: Abandoning Comfortable Blindness in Anaesthesia

**DOI:** 10.4274/TJAR.2026.262441

**Published:** 2026-02-09

**Authors:** Özlem Selvi Can, Alper Kılıçaslan, Serkan Tulgar, Zekeriyya Alanoğlu

**Affiliations:** 1 Ankara University Faculty of Medicine, Department of Anaesthesiology and Reanimation, Ankara, Türkiye; 2 Necmettin Erbakan University Faculty of Medicine, Department of Anaesthesiology and Reanimation, Konya, Türkiye; 3 Bahçeşehir University Faculty of Medicine, Göztepe Medical Park Hospital, Department of Anaesthesiology and Reanimation, İstanbul, Türkiye; 4 Washington University in St. Louis, School of Medicine, Department of Anaesthesiology St. Louis, United States

**Keywords:** Anaesthesia, cardiovascular and thoracic anaesthesia, comfortable blindness, contemporary anesthetic, patient safety

Defining patient safety in anaesthesia solely by keeping vital signs within “acceptable” limits on a monitor is no longer a scientific strategy; it has become a form of comfortable blindness. Although strong evidence shows that both the duration and depth of perioperative hypotension are linked to cardiac, renal, and neurological complications, hemodynamic disturbances, particularly those occurring during regional anaesthesia or sedation, are still often tolerated. This paradox remains one of the most striking inconsistencies in contemporary anesthetic practice.^1^

A similar problem exists in brain monitoring. Depth-of-anaesthesia monitors and processed electroencephalography (EEG) are now widely available, yet the information they generate does not always translate into meaningful clinical decisions. In elderly and frail patients, the relationship between burst suppression and postoperative delirium or neurocognitive dysfunction is well established.^2^ Even so, excessively deep anaesthesia may pass unnoticed behind screens that appear reassuringly stable.

The study by Çalışkan et al.,^3^ published in this issue, reminds us that such passivity is not inevitable. Combining structured perioperative care based on Enhanced Recovery After Surgery principles with the Safe Brain Initiative was associated with shorter hospital stays, improved pain control, and reduced incidence of postoperative cognitive dysfunction. These results suggest that patient-centered care and advanced monitoring are not luxuries but active components of treatment that directly shape outcomes.

Yet, two national surveys published in the this issue show that advanced hemodynamic and neurophysiological monitoring is still used sparingly during high-risk surgery.^4, 5^ Cost, limited access to equipment, and insufficient training are often cited as barriers. However, these explanations do not justify choosing not to look when the risks are already known. The problem is not so much the absence of monitors as the reluctance to treat the information they provide as a clinical responsibility.

Ultimately, the value of monitoring in modern anaesthesia does not lie in the technology itself. It lies in the clinician’s ability to interpret the data, place it in context, and translate it into individualized decisions. From this perspective, the limits of monitoring are conceptual rather than technical and are defined by clinical judgment.

Professional societies increasingly recommend tools such as processed EEG, cerebral oximetry, and goal-directed hemodynamic strategies for selected high-risk patients, yet consistent implementation remains limited. Protocols differ widely between institutions and practitioners, and the evidence regarding their effects on hard clinical outcomes continues to evolve.

Protection of the brain, the primary target organ of anaesthesia, cannot rely solely on hypnotic depth. Hemodynamic stability, cerebral perfusion, and neurophysiological integrity must be considered together. The question facing modern anaesthesia is whether we will choose to make the invisible visible or remain satisfied with what the screen shows and overlook what truly matters.
